# Nonsize Criteria for Surgical Intervention on the Ascending Thoracic Aorta

**DOI:** 10.1055/s-0043-1766114

**Published:** 2023-05-12

**Authors:** John A. Elefteriades, Bulat A. Ziganshin, Mohammad A. Zafar

**Affiliations:** 1 Aortic Institute at Yale-New Haven, Yale University School of Medicine, New Haven, Connecticut

**Keywords:** aorta, diameter, aortic surgery, thoracic aorta, ascending aorta, chest pain

## Abstract

For decades, aortic surgery has relied on size criteria for intervention on the ascending aorta. While diameter has served well, diameter alone falls short of an ideal criterion. Herein, we examine the potential application of other, nondiameter criteria in aortic decision-making. These findings are summarized in this review. We have conducted multiple investigations of specific alternate nonsize criteria by leveraging our extensive database, which includes complete, verified anatomic, clinical, and mortality data on 2,501 patients with thoracic aortic aneurysm (TAA) and dissections (198 Type A, 201 Type B, and 2102 TAAs). We examined 14 potential intervention criteria. Each substudy had its own specific methodology, reported individually in the literature. The overall findings of these studies are presented here, with a special emphasis on how the findings can be incorporated into enhanced aortic decision-making—above and beyond sheer diameter. The following nondiameter criteria have been found useful in decision-making regarding surgical intervention. (1) Pain: In the absence of other specific cause, substernal chest pain mandates surgery. Well-developed afferent neural pathways carry warning signals to the brain. (2) Aortic length/tortuosity: Length is emerging as a mildly better predictor of impending events than diameter. (3) Genes: Specific genetic aberrations provide a powerful predictor of aortic behavior; malignant genetic variants obligate earlier surgery. (4) Family history: Aortic events closely follow those in relatives with a threefold increase in likelihood of aortic dissection for other family members once an index family dissection has occurred. (5) Bicuspid aortic valve: Previously thought to increase aortic risk (as a “Marfan light” situation), current data show that bicuspid valve is not a predictor of higher risk. (6) Diabetes actually protects against aortic events, via mural thickening and fibrosis. (7) Biomarkers: A specialized “RNA signature test” identifies aneurysm-bearing patients in the general population and promises to predict impending dissection. (8) Aortic stress: Blood pressure (BP) elevation from anxiety/exertion precipitates dissection, especially with high-intensity weightlifting. (9) Root dilatation imposes higher dissection risk than supracoronary ascending aneurysm. (10) Inflammation on positron emission tomography (PET) imaging implies high rupture risk and merits surgical intervention. (11) A *KIF6* p.Trp719Arg variant elevates aortic dissection risk nearly two-fold. (12) Female sex confers some increased risk, which can be largely accommodated by using body-size-based nomograms (especially height nomograms). (13) Fluoroquinolones predispose to catastrophic dissection events and should be avoided rigorously in aneurysm patients. (14) Advancing age makes the aorta more vulnerable, increasing likelihood of dissection. In conclusion, nondiameter criteria can beneficially be brought to bear on the decision to observe or operate on specific TAA.

## Introduction

Although having previously touched on some concepts that may fall under the rubric of “nondiameter” criteria for intervention on the ascending thoracic aorta, 1 our team at the Aortic Institute at Yale had not previously organized intervention criteria under the concept of “nondiameter” factors. An invitation from Drs. Mark Field and Manoj Kuduvalli to deliver an invited lecture on this topic at the Liverpool Aortic Symposium 2022 stimulated our intensive look at nondimensional predictors of aortic behavior, which form the substance of the present study.

Nonsize factors that weigh in the decision to operate on the thoracic aorta include the following:

- Pain.
- Length/tortuosity.
- Genes.
- Family History.
- Bicuspid aortic valve.
- Diabetes.
- Biomarkers (“RNA signature”).
- Aortic Stress (exercise, blood pressure [BP]).
- Root location of dilatation.
- Inflammation (positron emission tomography [PET] imaging).
- KIF6 (Kinesin family member 6) genetic variant.
- Female sex.
- Fluoroquinolone Rx.
- Age.

We will discuss each of these factors individually in the following sections.

## Pain

**Fig. 1:**
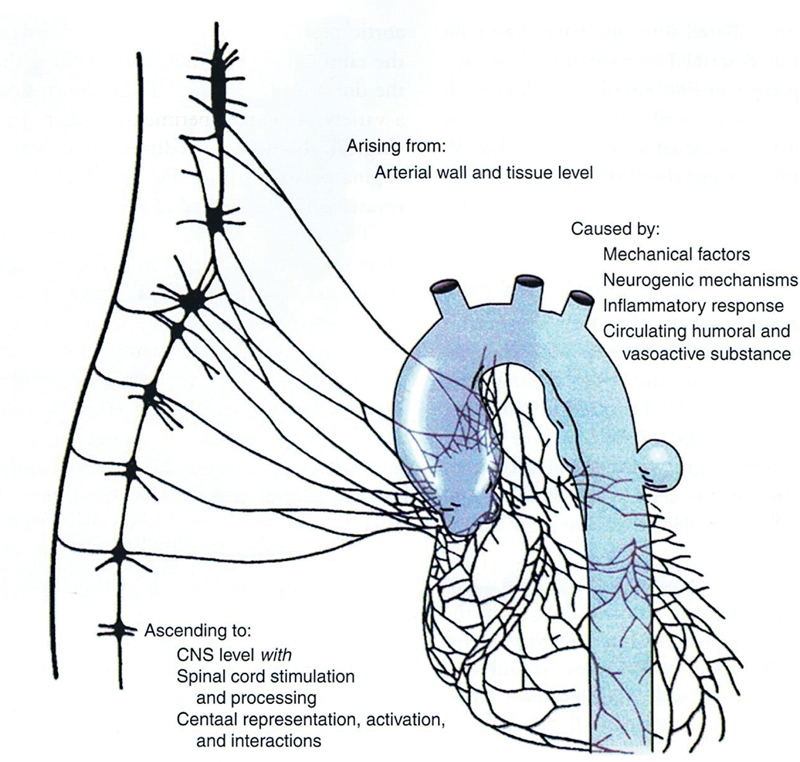
The pain pathway from the aorta to the brain (CNS, central nervous system). (Reproduced with permission from Wooley CF, Sparks EH, Boudoulas, Aortic Pain. In: Boudoulas H, Stefanadis C, eds. The Aorta: Structure, Function, Dysfunction, and Diseases. New York, NY: Informa; 2009.)

As can be seen in Fig. 1 , the aorta is invested by a network of afferent pain fibers, which transmit noxious sensations via the sympathetic chain to the brain. Not all precordial chest pain originates in the myocardium. As emphasized by Boudoulas and Stafanadis, the aorta should always be kept in mind as a potential source of chest pain. 2 Pain is the only language in which the aorta can speak to us. Most importantly, if a patient with a known ascending aortic aneurysm develops chest pain, serious consideration should be given to resection of that aneurysm. Simply put, a painful aneurysm needs to be taken very seriously and likely merits resection, regardless of size. 3 4 5

**Fig. 2:**
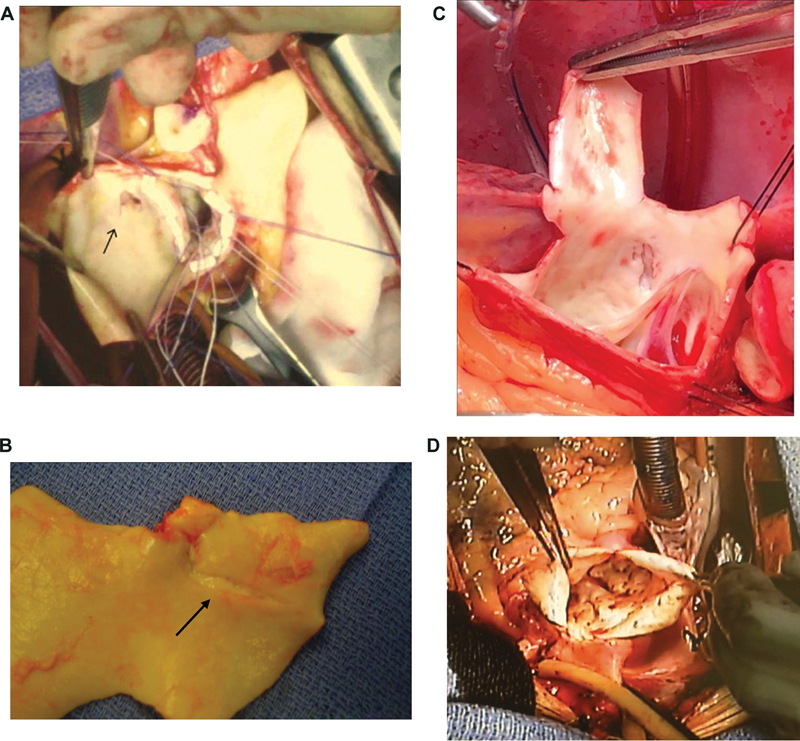
Operative findings in patients with pain but without enlargement of the ascending aorta to dimensions meeting the surgical criteria. These patients were all operated for pain, specifically. ( **A–C** ) Clear-cut aortic dissections that halted spontaneously, without propagation downstream in the aorta. ( **D** ) Innumerable penetrations deep into the aortic wall. Image courtesy: Dr. John Elefteriades, MD (personal photos).

Fig. 2 demonstrates the endoluminal aortic findings in patients who underwent surgery for pain rather than for aortic size. One can see dramatic disruptions of the aortic intima, with cavitary lesions penetrating into the media of the aorta. Fig. 2A–C shows aortic dissections that stopped their progress spontaneously but continued to cause ongoing pain. Fig. 2C was kindly contributed by Dr. Duke Cameron, to emphasize the importance with which pain is considered in his practice as well. Fig. 2D shows a virulent destructive process, with innumerable penetrations into the media, none of which had been recognized on preoperative imaging in this patient operated for pain.

## Length/Tortuosity

In terms of aortic dimensions, attention has been nearly universally focused on *diameter* , largely ignoring other aortic size characteristics. We have referred to aortic length as “the neglected dimension.” Recently, attention has been turned to aortic length and its correlate, aortic tortuosity. As the aorta lengthens, it needs to become tortuous (or “curved”) to remain within its body confines. Although one could consider length a “size” measurement, we are including length in this manuscript because it extends analysis beyond diameter and, by recent evidence, appears to be of cardinal importance.

**Fig. 3:**
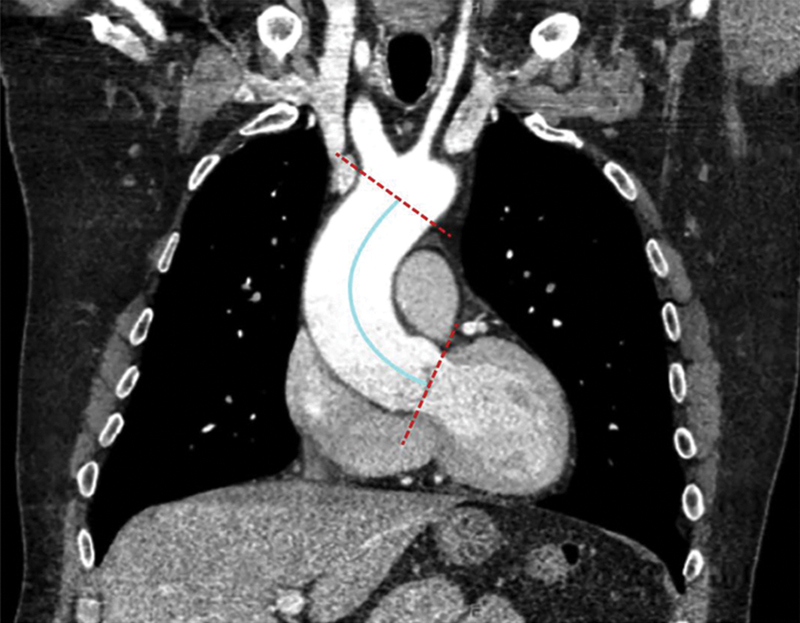
Aortic length, defined as the centerline length from the aortic annulus to the base of the innominate artery. “Red” lines indicate annulus and origin of the innominate artery and “blue” line indicates “length”. (Reproduced with permission from Wu et al. 6 )

**Fig. 4:**
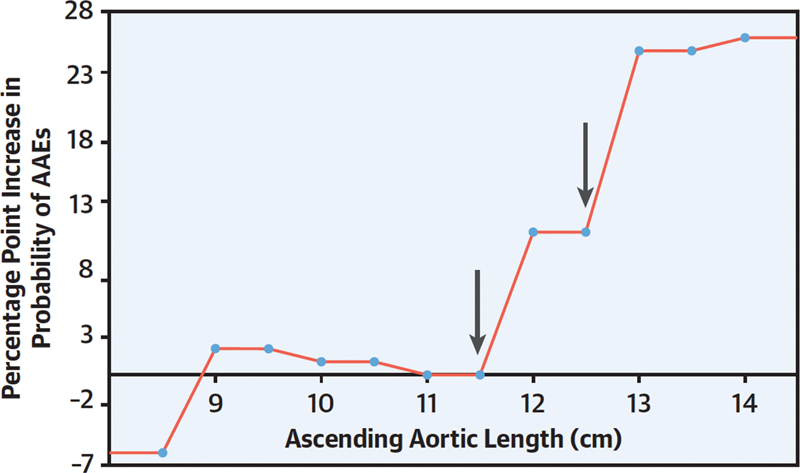
Note that lengthening of the ascending aorta to 13 cm is critical, incurring a very high risk of adverse aortic events (AAEs). Note also that there are two earlier “hinge points” at which risk rises incrementally, at 11.5 cm and at 12.5 cm. (Reproduced with permission from Wu et al. 6 )

**Fig. 5:**
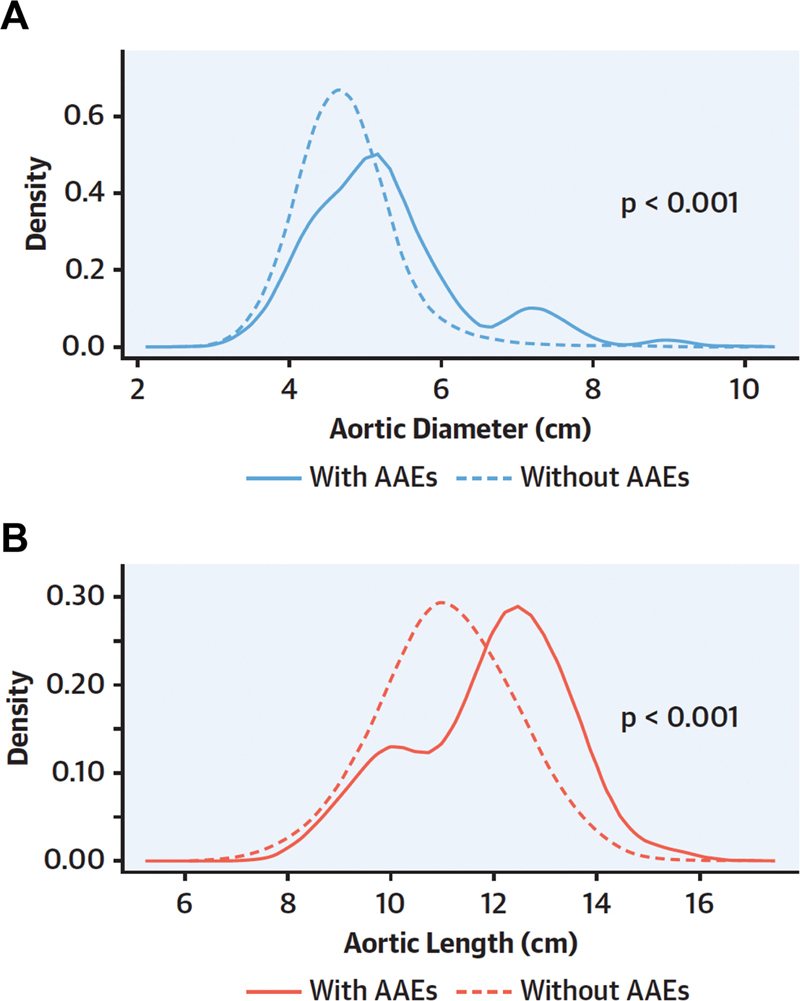
Herein are displayed the “density” curves of aortic size (i.e., the proportion of patients at different size ranges). ( **A** ) It indicates curves for diameter as a criterion, and ( **B** ) indicates length. Note that the aortic length better separates the curves of patients with versus without adverse aortic events (AAEs) than does diameter. (Reproduced with permission from Wu et al. 6 )

**Fig. 6:**
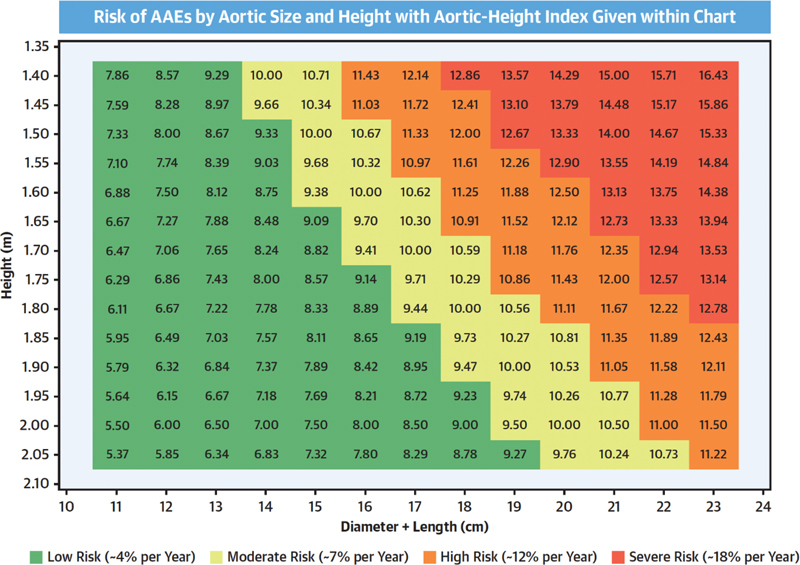
Aortic height index (AHI) nomogram as a function of aortic size (diameter + length; x-axis) and patient height (y-axis), with the AHI given within cells. The table has a four-tier, color-coded warning system, with *red* representing the most severe, followed by orange, yellow, and green. AAEs, aortic adverse events. (Reproduced with permission from Wu et al. 6 )

We and others have chosen to measure (or “define”) the ascending aortic length as the distance from the aortic annulus to the base of the innominate artery (see Fig. 3 ). Both landmarks, the annulus and the base of the innominate artery, are clearly defined structures. While diameter is a good predictor of aortic adverse events, we have recently found that length is even a notch better prognostically. 6 As shown in Fig. 4 , aortic lengthening to >13 cm signifies an extremely high risk of aortic dissection. Others have noted similar findings. 7 8 9 In fact, certain metrics show that ascending aortic length is an even better predictor than diameter. This is shown in Fig. 5 , where we connote somewhat greater discrimination between patients free from or patients incurring adverse aortic events (AAEs). We found, favorably, that length (unlike diameter) does not change substantially at the moment of dissection, rendering length preferable in this regard. 6 We do not wish to denigrate diameter in any way; however, ascending aortic length is even a bit better predictor. We all need to incorporate ascending aortic length more fully in our day-to-day clinical assessments. We publish a nomogram using aortic length to predict expected risk of aortic events ( Fig. 6 ).

## Genes

Once the familial nature of thoracic aortic aneurysm and dissection (TAAD) was documented in the late 1990s, 10 11 scientists have been on the hunt to identify the specific genes that cause this disease. Currently more than 60 genes have been implicated as being potentially causative of TAAD, 12 although only 24 of these have passed the ClinGen curation (with only 11 genes in the definitive or strong evidence category). 13 These genes can cause either syndromic or nonsyndromic TAAD, or both. 14 These genes encode regulatory molecules for the extracellular matrix ( *FBN1* , *FBN2* , *COL1A1* , *COL1A2* , and *COL3A1* ), the cytoskeleton in smooth muscle cells ( *ACTA2* , *MYH11* , and *MYLK* ), and the TGF-β signaling pathway ( *TGFβ2* , *TGFBR1* , *TGFBR2* , *SMAD3* , and *SLC2A10* ). 14

It is now well recognized that certain genes can alter the natural course of aortic disease by making patients more vulnerable to AAEs (such as rupture and dissection) at small aortic sizes, 15 16 well below the traditional intervention criteria, 17 18 and at a significantly younger age. This was first recognized for patients with mutations in the *FBN1* gene (Marfan's syndrome), for whom the intervention criteria were lowered in the the American and European Aortic Disease guidelines. 18 19 Additional genes, for syndromic or nonsyndromic thoracic aortic aneurysm (TAA), also predispose to early clinical events. For example, mutations in the *MYLK* gene predispose patients exclusively to aortic dissection, 20 while mutations in in the *ACTA2* gene cause aortic dissection at small aortic sizes. 21 22

So it is vital for the clinician to take these genetic variants into account in determining criteria for surgical intervention on the ascending aorta in specific patients in whom this detailed genetic information is available.

**Fig. 7:**
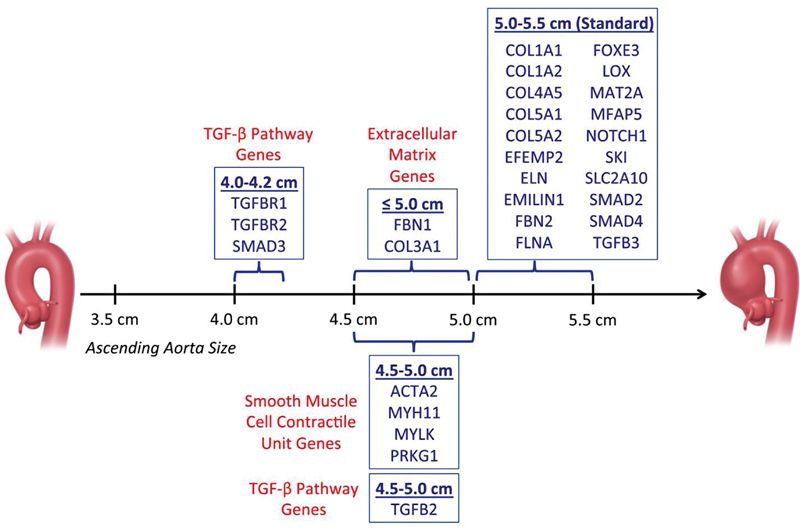
“Timeline” for intervention, demonstrating current criteria for intervention for common genetically triggered thoracic aortic aneurysms. (Reproduced with permission from Brownstein et al. 23 )

**Fig. 8:**
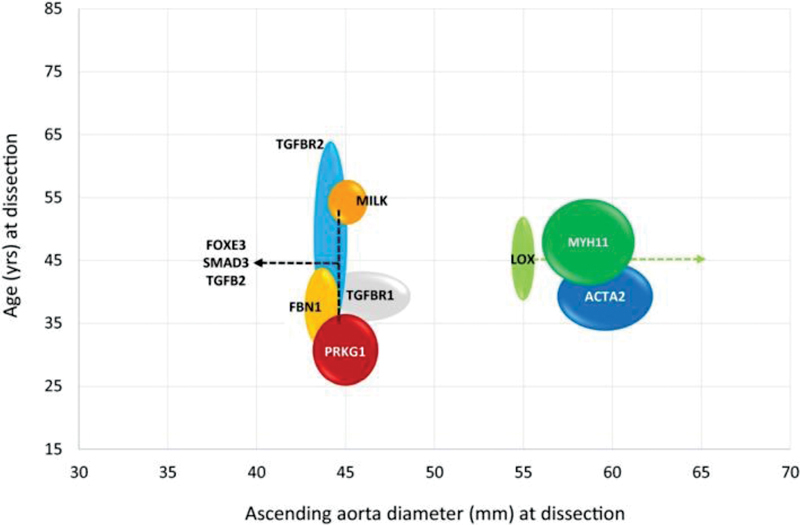
Schematic representation of both age and aortic diameter for patients with specific causative genetic mutations. This can aid clinicians in the decision to operate on such patients. (Reproduced with permission from Mariscalco et al. 25 )

To aid clinicians with decision-making in the setting of a pathogenic variant in a particular gene, we publish and regularly update recommended intervention thresholds for each of the currently known TAAD genes (see Fig. 7 ). 23 24 Please note that many genes currently listed at the “Standard (5.0–5.5 cm)” may need to move “earlier” as more clinical data accumulate. Further guidance for surgical intervention is provided by Mariscalco et al, 25 who plot patient age versus diameter, both at the time of an aortic dissection event; they do this for several common genes that predispose to TAAD (see Fig. 8 ). This analysis can help the clinician considerably by incorporating the very important age parameter in decision-making.

In summary, knowing which gene is the culprit for TAAD in a particular patient is a critical criterion in the decision to operate.

## Family History

Another important and often overlooked factor that should influence surgical intervention on the ascending aorta is family history. Once a patient has been identified as having thoracic aortic disease (aneurysm or dissection), it is critical to screen family members, who may be asymptomatic but still harbor an aneurysm. We screen very widely: parents, children, siblings, aunts, uncles, nieces and nephews, and grandchildren. An echocardiogram usually suffices as a screening tool, although it is usually unreliable in visualizing the aorta beyond the mid-ascending portion.

At our Aortic Institute at Yale, working together with Dr. Michael Coady, we determined that familial aneurysms are more malignant than sporadic aneurysms, presenting at a younger age (58 vs. 66 years), and growing faster (2.1 vs. 1.6 mm/y). 26 Thus, earlier surgical intervention may be necessary to prevent aortic dissection and rupture. However, if a patient has a family history of an aortic dissection event, this is an even more powerful predictor of malignant outcome, increasing the odds or rupture of dissection in family members by a massive three- to ninefold. 27 28 29 Furthermore, familial aortic dissection events tend to cluster in regard to the age of dissection onset: at least 50% of patients will develop an aortic dissection within 5 years (younger or older) of the age at which the initial dissection event in the family occurred. 30

So family history is vitally important as a criterion for surgical intervention. It has been our policy to offer surgical intervention to any patient with a “real” aneurysm (say 4.2 cm and above) who also has a family history of aortic dissection.

## Bicuspid Aortic Valve

**Fig. 9:**
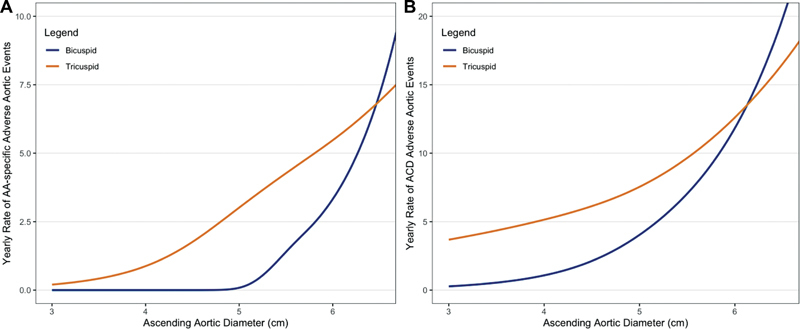
Comparison of event rates in patients with ascending aortic aneurysms in the presence of bicuspid aortic valves (blue) and trileaflet aortic valves (orange). The left graph shows ascending aorta (AA) specific events; the right graph shows all-cause death (ACD) adverse events. In both analyses, patients with bicuspid aortic valves are shown to be safer than those with trileaflet valves. Submitted for publication. Reprinted with permission from Zafar et al. 31

For decades, it was thought that aneurysms associated with bicuspid aortic valve were highly dangerous, almost as much as the aortas of patients with Marfan's disease. In fact, the term “Marfan's light” became popular to describe the increased susceptibility of the aorta in patients with bicuspid aortic valve. A recent analysis from our center has found that this is actually not the case. In fact, the aneurysmal ascending aorta in patients with bicuspid aortic valve is no more vulnerable than that of patients with a normal, trileaflet aortic valve (see Fig. 9 ). 31 The American Association for Thoracic Surgery consensus “Guidelines on bicuspid aortic valve-related aortopathy” agree with our findings, stating specifically that “An increasing amount of literature has recently shown that BAV (bicuspid aortic valve)-related aortopathy is less dangerous than previously described.” 32 Therefore, ascending aortic aneurysm patients with bicuspid aortic valve do not need to be operated sooner or at smaller dimensions than other patients.

## Diabetes

Diabetes has a long-standing, well-earned reputation as a profoundly negative health factor. That is why it is so frankly astounding that diabetes seems, along nearly every avenue of analysis, to play a protective role in patients with aneurysm disease. Our team recently reviewed the literature on this topic. 33

**Fig. 10:**
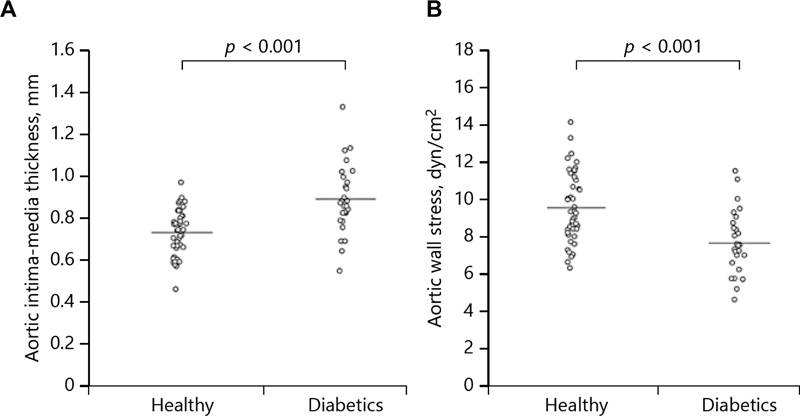
( **A** ) Note thicker wall in diabetics, translating into ( **B** ) lower wall stress. (Reproduced with permission from Astrand et al. 34 )

First, diabetes increases the thickness of the aortic wall. Surgeons know this from experience, but this has been shown scientifically as well 34 (see Fig. 10A ). Specifically, wall thickness in the figure is seen to be substantially higher in diabetics than in nondiabetics. Because wall thickness appears in the denominator of Laplace's law ( *T* = *P* × *r* / 2 × *t* ), where *T* is wall tension, *P* is intraluminal pressure, *r* is radius, and *t* is thickness, wall tension is decreased, as seen in Fig. 10B . Decreased wall tension is very beneficial in an aneurysmal wall.

**Fig. 11:**
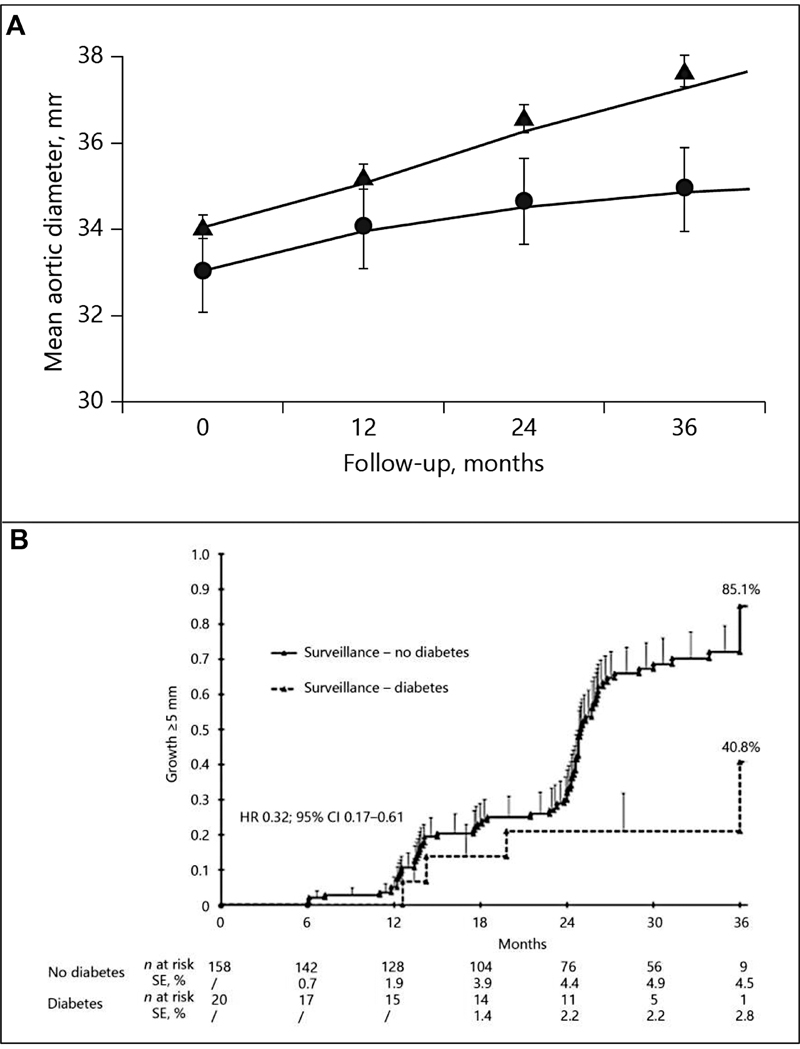
Note slower growth rate of diabetic aortas (circles) ( **A** ) and ( **B** ) much higher percentage of nondiabetic aortas reaching a growth rate of ≥5 mm. (Reproduced with permission of Golledge et al 35 and De Rango et al 36 )

So, there are engineering benefits in the aortic wall. Do these translate to observable clinical benefit? We see in Fig. 11A that expansion rate was less in the diabetic aortas (circles). 35 In Fig. 11B , we see that far more nondiabetics than diabetics achieved an aortic growth rate of ≥5 cm during observation. 36

Studies have shown, and clinical experience in the operating room confirms, that the diabetic aorta is grossly thickened, dense, and fibrotic, and holds sutures well. The diabetic aorta is paradoxically protective against both abdominal aortic aneurysm (AAA) and TAA. Furthermore, the diabetic aorta is inimitable to aortic dissection.

Although most studies have been done in AAA and fewer in TAA, experiments have shown all the following effects of diabetes in aortic aneurysm disease: decreased incidence and prevalence, decreased aneurysm growth rate, lower matrix metalloproteinase (MMP) levels, decelerated matrix loss, decreased dissection rate and rupture rate, decreased mortality, and delayed age at rupture. 33 37 38 39 40 41 42

This section has discussed the beneficial impact of diabetes itself. It is worth noting that the drug metformin, used so commonly in the treatment of diabetes, has in and of itself marked beneficial properties vis-à-vis aneurysm disease. 33 43 These salutary effects include protection against AAA and TAA and also decreased adverse factor levels (MMP2, MMP9, TNFa, and IL-6) in both mice and humans. Metformin also has cardioprotective and vascular protective effects—and it enhances weight loss as well.

So, paradoxically, diabetes, so detrimental in arteriosclerosis, has been found markedly beneficial from a purely aneurysm standpoint; the diabetic aorta, dense, thickened, and fibrous, has been found inimitable to aortic dissection. Also, the common medical treatment for diabetes, metformin, has been found to be additively beneficial as well.

## Biomarkers

One of the most serious problems in aortic disease has to do with the identification of asymptomatic carriers of aneurysm in the general population. Once an aneurysm-bearing patient is identified, medical and surgical science can keep that patient safe. We know reasonably well when to operate (although this article aims to refine those abilities), and we know how to operate. So, we need to improve identification of asymptomatic carriers in the general population.

We have performed a biomarker study, in which we assessed the circulating levels of 33,000 ribonucleic acids (RNAs). It should be remembered that deoxyribonucleic acid (DNA) is the blueprint for how the body will be made, but RNAs are the worker moieties that carry out the actual process of building the body. RNAs can be upregulated or downregulated depending on the needed activity in different organs and tissues.

**Fig. 12:**
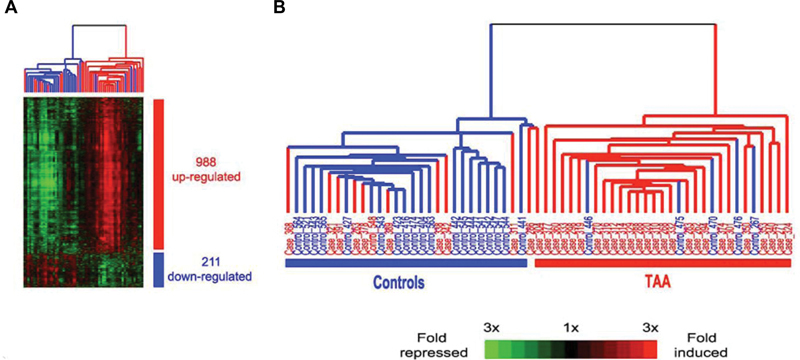
Results of screening of ribonucleic acid (RNA) expression levels of 33,000 RNAs in patients with and without thoracic aortic aneurysm. *Red* indicates overexpression of our panel of select genes, and blue indicates under-expression. If all the *reds* were together and all the blues were together, the test would have been 100% accurate. The test was encouragingly accurate at predicting correctly which patients harbored a thoracic aortic aneurysm (TAA) and which did not. Sensitivity was 83% and specificity was 68%, and the overall accuracy was 78%. (Reproduced with permission from Wang et al 44 )

We were able to identify, from among those 33,000 RNAs, a panel of expression levels of 41 genes that was quite effective at discriminating which patients harbored an aneurysm and which did not, with an overall accuracy approaching 80% ( Fig. 12 ). 44 We are just now replicating this work in a different cohort of patients. This promises to enable detection of asymptomatic patients harboring TAAs.

Another clinical issue has to do with anticipation of impending aortic dissection. Although many “biomarkers” for aortic dissection have been developed, these detect dissection after it has occurred. So those tests are not very helpful, as the dissection has already occurred, and imaging studies have generally confirmed aortic dissection by the time those biomarker results become available. D-dimer (essentially always positive when there is an aortic dissection, reflecting clot in the false lumen of the dissected aorta) is of some utility in the emergency department in questionable cases prior to echocardiography or computed tomography (CT) scanning. However, what is really needed is a biomarker that can indicate that an aortic dissection is looming, about to occur.

Within the 33,000 genes tested in the study described earlier, we were able to identify a subpanel of a dozen up- or downregulated genes that were able to distinguish patients at the moment they suffered aortic dissection from those free of dissection (unpublished). We speculate that the abnormal RNA profile, reflecting disordered aortic biology, was likely abnormal for some time before dissection occurred—making this RNA profile a potential monitoring test. While still in the early stages, this molecular-style approach holds promise for enhanced prediction of dissection based on specific activated or deactivated biological pathways. Such predictive markers of impending dissection could represent an important step forward in aortic care.

## Aortic Stress

**Fig. 13:**
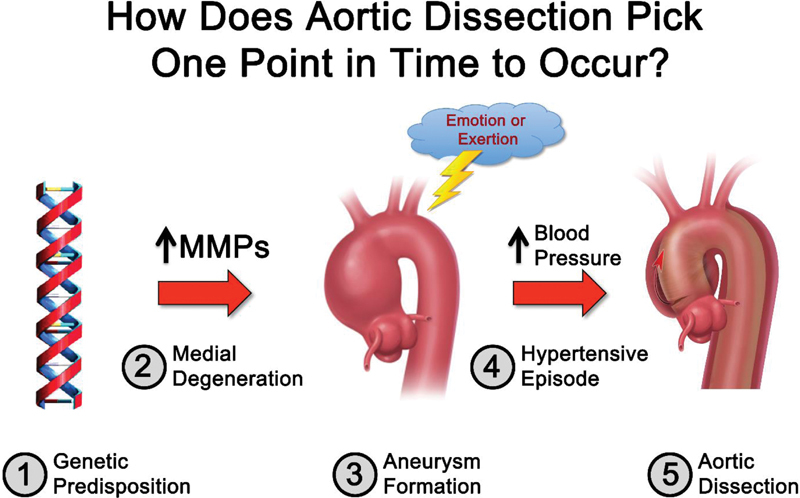
The biological pathway leading to aortic dissection (see text). MMP, matrix metalloproteinases. (Reproduced with permission from Hatzaras IS, Bible JE, Koullias GJ, Tranquilli M, Singh M, Elefteriades JA. Role of exertion or emotion as inciting events for acute aortic dissection. Am J Cardiol 2007;100:1470–1472.)

The level of mechanical stress imposed on the aorta has an important impact on the occurrence of aortic dissection. We have published the paradigm shown in Fig. 13 to represent the clinical path that eventuates in aortic dissection. This analysis is based on multiple studies we have performed and published over decades. 45 46 47 48 In brief, we feel that many, if not most, pathways toward dissection originate with a genetic mutation that predisposes to aortic disease. We currently find suspicious variants in about one-third of patients genetically sequenced. 49 This number increases as whole exome sequencing proliferates. We believe that the genetic abnormality, over decades, leads to destruction of the aortic wall elements (e.g., lamellar loss), thus weakening the wall and allowing its gradual enlargement over time—ultimately to aneurysmal dimensions. Then, we believe, an acute, severe hypertensive episode raises blood pressure and aortic wall tension beyond the tensile strength of the chronically weakened aortic wall—resulting in an acute aortic dissection event. Two-thirds of our patients recall a specific severe physical exertion or an especially troubling emotional event just before onset of their dissection pain.

So, aneurysm patients subject to severe physical exertion or severe emotional events should be considered dissection prone and triaged earlier (in time and in aortic size) to surgical therapy. This is an extremely important factor to take into account. (Of course, it is helpful to mitigate these physical and emotional stressors via advice and restrictions, but compliance is often marginal). We do recommend a B-blocker and an afterload reducing drug for most patients, to “take the edge off” hypertensive episodes.

**Fig. 14:**
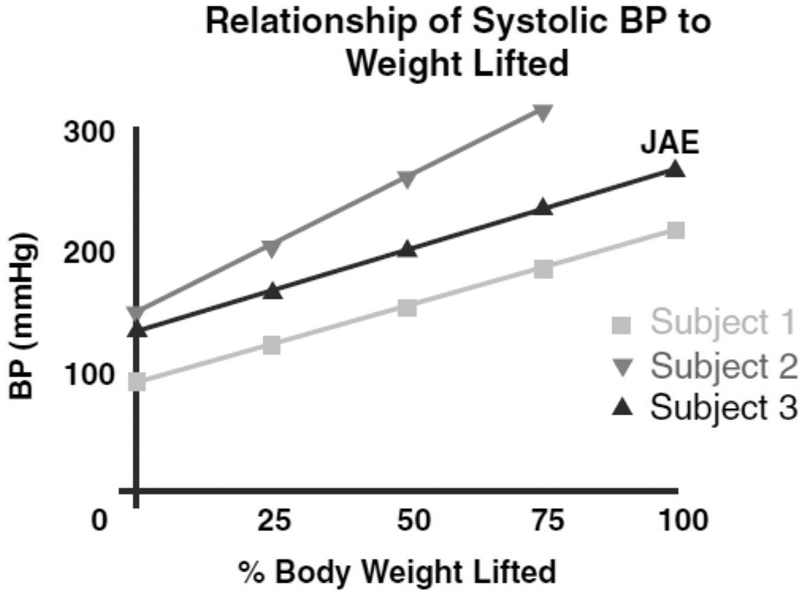
Note the extreme levels of hypertension reached during the effort cycle of the bench press exercise. Blood pressure was measured with a bespoke apparatus capable of detecting momentary rises in blood pressure, much too short in duration to be measured by a cuff device. Subject 1: a healthy 14-year-old athlete. Subject 2: a healthy 48-year-old man, weightlifting since age 12. Subject 3: a healthy 57-year-old former athlete, now sedentary. Note the pressure of 320 mm Hg reached by Subject 3, lifting 75% of his body weight, when his exercising was stopped due to the absolute magnitude of the pressure obtained. (Reproduced with permission from Elefteriades. 47 )

Weightlifters are especially vulnerable, as blood pressure reaches astronomical levels (not seen in any other human setting or endeavor) during the effort cycle of the “lift” (see Fig. 14 ). We know of no other environment, in the outside world or within the hospital, where absolute magnitude blood pressures over 300 mm Hg are achieved. Table 1 presents dozens of cases made known to our team of exercise-induced aortic dissection—a very real and at least partially preventable cause for the loss of promising young (and older) athletes.

**Table 1:** Synopses of cases sent to the senior author manifesting exercise-induced aortic dissection, often lethal

No.	Occupation	Age (y)	Sex	Treatment site	Family history	Activity	Aortic size (cm)	Type of dissection (ascending or descending)	Surgery	Outcome
1	Student	24	M	Yale	Yes	Weightlifting	5.5	Ascending	Yes	Alive
2	Student	19	M		No	Weightlifting	5	Ascending	No	Dead a
3	Salesman	53	M	Yale	No	Weightlifting	4	Ascending	Yes	Alive
4	Policeman	37	M	Yale	No	Pushups	5	Ascending	Yes	Alive
5	Security	52	M		No	Pushups		Ascending	No	Dead b
6	Attorney	68	M		No	Weightlifting (175 Ib)	“Dilated”	Ascending	Yes	Dead
7	Signalman	55	M		No	Lifting generator (80 Ib)	3	Ascending	Yes	Dead
8	Repairman	44	M		No	Lifting tank (400 Ib)	7.8	Ascending	Yes	Alive
9	Professor	49	M		No	Weightlifting	6.3	Ascending	Yes	Alive
10	Writer	43	M		No	Weightlifting (300 Ib)		Ascending	No	Dead b
11	Social worker	42	M		No	Weightlifting	4	Ascending	Yes	Alive
12	Surgeon	63	M		Yes	Weightlifting	3.8	Descending	Yes	Alive
13	Mason	34	M		No	Lifting concrete blocks (150 Ib)	4	Descending	No	Alive
14	Priest	56	M		No	Weightlifting (250 Ib)	3	Ascending	Yes	Alive
15	Businessman	40	M		No	Weightlifting	6.9	Ascending	Yes	Alive
16	Journalist	50	M		No	Weightlifting (500 Ib)		Ascending	Yes	Alive
17	Surgeon	43	M		No	Intense swimming	4	Ascending	Ves	Alive
18	Mason	75	M	Yale	No	Intense swimming	6	Ascending	Yes	Alive
19	Clerk	49	F	Yale	No	Pulling hard against heavy weight	4.3	Ascending	Yes	Alive
20	Professor	74	M	Yale	No	Intense tennis	4	Descending	Yes	Alive
21	Mailman-ret	76	M	Yale	No	Moving heavy boxes	4.3	Descending	Yes	Alive
22	Unemployed	35	M	Yale	No	Exercising	3.1	Ascending	Yes	Alive
23	Computers	50	M	Yale	No	Changing storm windows	6	Ascending	Yes	Dead
24	Security guard	48	M	Yale	No	Intense swimming	4.9	Ascending	Yes	Alive
25	Businessman	35	M	Yale	No	Intense racquetball	4.1	Ascending	Yes	Alive
26	Machinist	50	M	Yale	No	Shoveling snow		Ascending	Yes	Alive c
27	Mechanic	51	M	Yale	Yes	Weightlifting	6	Ascending	Yes	Alive c
28	–	37	M		No	Weightlifting		Ascending	No	Dead b
29	Construction	35	M		No	Lifted power washer from truck	4.1	Ascending	No	Dead b
30	Mover	38	M		No	Carried freezer 2 flights (700 Ib)	4.3	Ascending	No	Dead b
31	Engineer	43	M		No	Weightlifting	4	Asceding	Yes	Dead

Source: Reproduced with permission from Hatzaras et al. 46

a Diagnosis made by imaging (echocardiography or computed tomography [CT]), but the patient was not transferred in time for surgery.

b Diagnosis not made during life. Postmortem confirmatory.

c Prior Type B dissection.

## Root Location of Dilatation

In the past, we in cardiac surgery did not adequately differentiate risk levels for given diameters depending on whether they were located in the ascending aorta proper or on the aortic root. One of our team members has expertly sorted out these risks for the two zones: the ascending aorta above the sinotubular junction versus the aortic root itself.

**Fig. 15:**
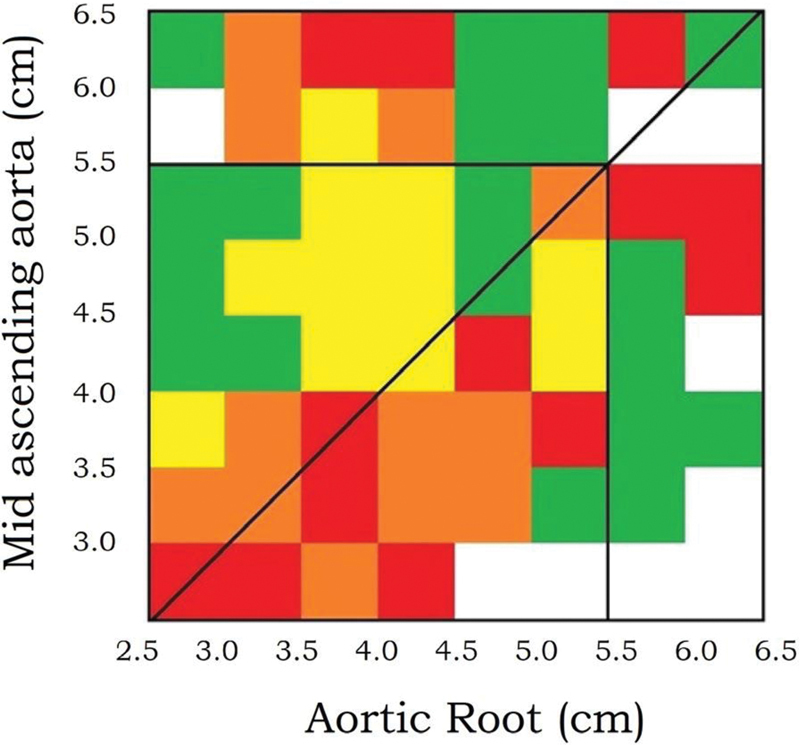
Risk matrix for 1,162 patients. The risk of the composite end point for each combination of aortic root and mid-ascending aorta size is expressed on a color scale with *green* as low risk (<5%), *yellow* as intermediate risk (5–10%), *orange* as high risk (10–20%), and *red* as very high risk (>20%). Above the oblique line, the mid is more dilated than the root. Below the oblique line, the opposite is true. The matrix portrays considerable risk for both root and mid-dilatation. However, its coloring conveys at first glance that root dilatation is more malignant than mid-dilatation. The *vertical and horizontal black lines* inside the matrix outline the surgical threshold of 5.5 cm; therefore, the top 2 rows and the far right 2 columns are biased because of surgery. Note the concentration of more malignant behavior below the *diagonal black line* , indicating the severity of root dilatation. The right lower corner of the grid is empty, because we operated on patients in our sphere with root dilatation in those ranges, not allowing them to go on fully to an adverse clinical event. (Reproduced with permission from Kalogerakos et al. 50 )

Dr. Kalogerakos and colleagues performed a precise risk analysis for these two zones. Root dilatation was more malignant, producing more adverse events at a given level of enlargement. Earlier intervention for root dilatation than pure ascending dilatation was recommended 50 (see Fig. 15 ). Very recent data from the International Registry of Acute Aortic Dissection (IRAD) is supportive of this concept. 51

## Inflammation (Positron Emission Tomography Imaging)

A career's worth of data have been accumulated by Sakalihasan et al 52 confirming that the inflammation that is known to underlie aneurysmal degeneration of the aortic wall (driving the degeneration, in fact) can be visualized as “hot” uptake on PET of the chest or abdomen. Hot spots are seen more commonly in the abdominal aorta than in the ascending or descending thoracic aorta. Sakalihasan et al 52 have shown that such “lighting up” on PET presages adverse events and constitutes a strong indication for surgical intervention. 53

## KIF6

**Fig. 16:**
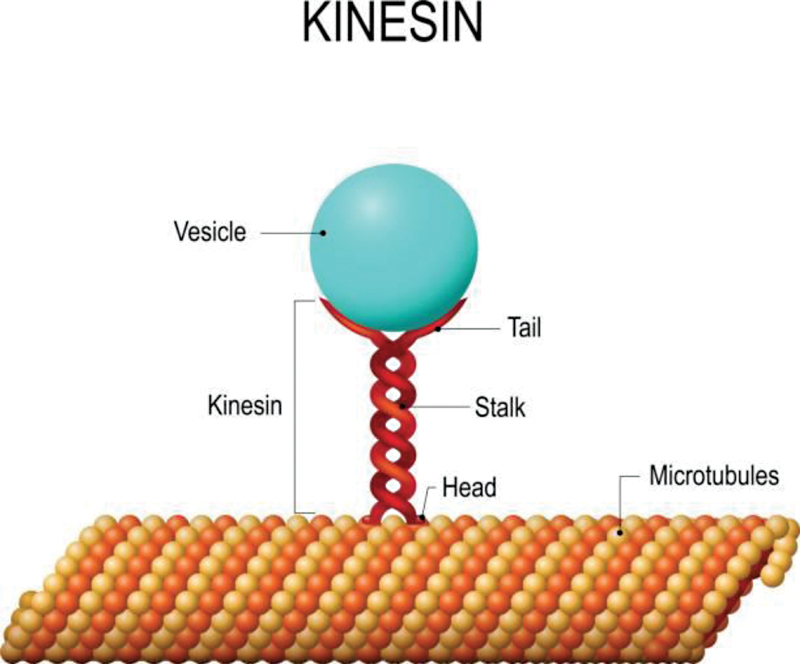
Kinesin “walks” vesicles along microtubules toward the nucleus of the cell. Free access image ( *https://www.istockphoto.com/photos/microtubule* ).

KIF6, a protein encoded by the gene of the same name, serves many important intracellular functions, including the transfer of vesicles and organelles from the periphery of the cell along microtubules toward the nucleus of the cell (see Fig. 16 ). Experiments in zebrafish have demonstrated that variants of this gene result in scoliosis. 54 Spinal abnormalities, of course, are part and parcel of multiple thoracic aortic syndromes. *KIF6* testing was commonly used several years ago to predict statin responsiveness. This test is still available via Quest Laboratories. Together with Dr. Olga Iakoubouva, an expert in *KIF6* (especially its relation to atherosclerosis and statin therapy), we performed a pilot project on *KIF6* , finding that carriers of the 719Arg variant of *KIF6* were more prone to TAA. 55 We have recently completed a large-scale investigation of the impact of *KIF6* on TAA, finding that harboring even a single variant dramatically increases the odds of aortic dissection (odds ratio [OR] ∼1.7–2.0 for various categories of dissection). 56 We are excited that ordering assays of KIF6 for our TAA patients may allow us to estimate the likelihood of dissection events with greater precision than before.

## Sex

Female sex raises risk of ascending aortic events mildly, but this is well accommodated by body size indexing, as in the height-based nomogram depicted in Fig. 6 .

## Fluoroquinolone Rx

Over recent years, through the precise work of LeMaire et al 57 and others, it has become abundantly clear that fluoroquinolone treatment disrupts the aorta and leads to aortic dissection in unfortunate patients. 58 This adverse relationship has been shown both in humans and in animal models. The Food and Drug Administration (FDA) has added a warning to the drug label. The dissection event may occur early or late after treatment and after small or large dosages of the drug. Eliciting a fluoroquinolone history has become an important part of aneurysm patient encounters. Fluoroquinolones should be fully avoided in patients with aortic disease.

## Age

**Fig. 17:**
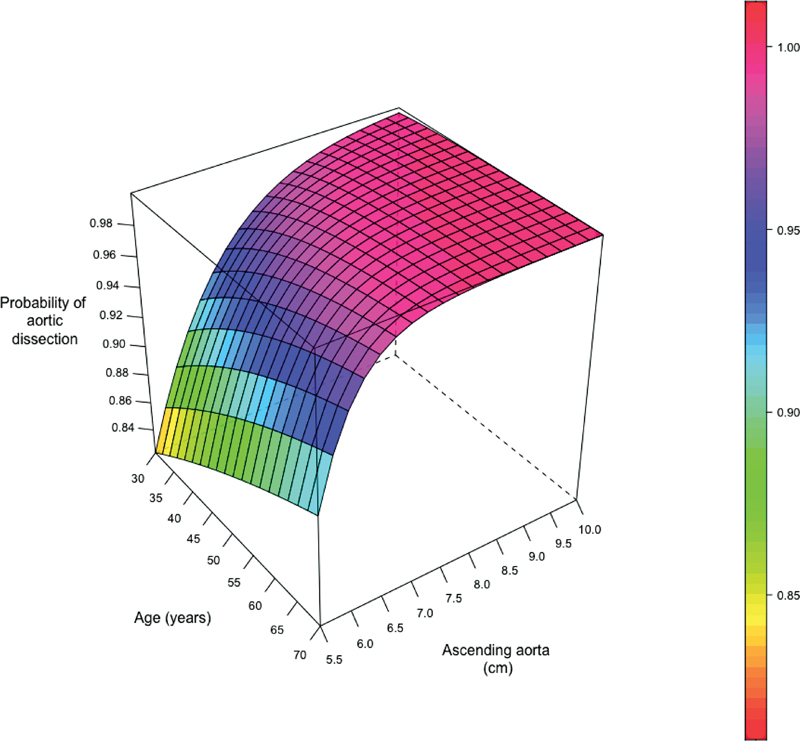
The probability of aortic dissection at various diameters increases with increasing patient age, reflecting the adverse impact of age (wear and tear) on the aortic wall.

Finally, as can be seen in Fig. 17 , the likelihood of aortic dissection occurring at a specific aortic diameter increases with increasing age, reflecting the “wear and tear” aging of the elements of the aortic wall. Serial studies of aortas of various ages have shown age-related deterioration—with thickening of the endothelium, thinning of the media, and loss of elastin. 59 60 61 Such changes make the aortic wall more susceptible to aortic dissection. This aging factor needs to be incorporated in our assessment of risks.

## Conclusion

**Fig. 18:**
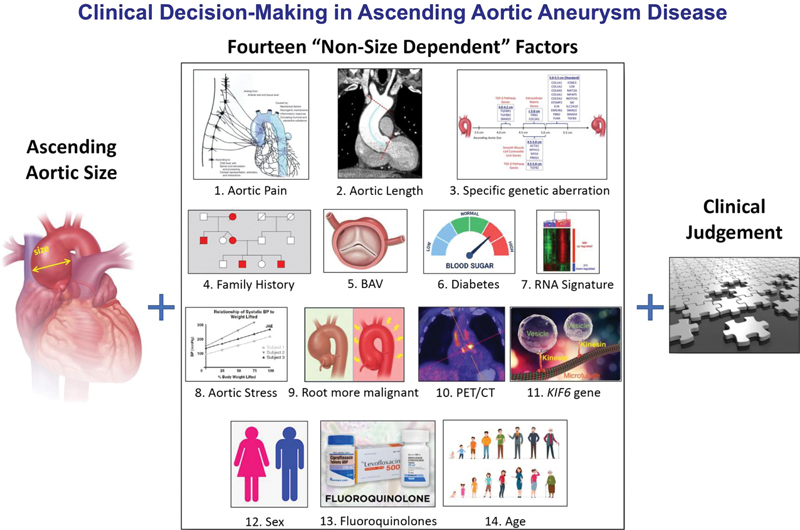
Summary graphic illustrating clinical decision-making in ascending aortic aneurysm disease.

Diameter has served our profession well as a criterion for surgical intervention for ascending aortic aneurysms. However, diameter alone is far from perfect as a predictor. We have outlined here 14 additional criteria that clinicians can apply to fine-tune their risk strategy for ascending aneurysm patients ( Fig. 18 ). Most of these factors increase risk: pain, excess aortic length, genetic abnormalities, family history, excess aortic stress, root location, and inflammation. Bicuspid aortic valve, we have shown, is neutral. Biomarker analysis is still evolving, and the presence of diabetes substantially lowers risks. Table 2 summarizes these findings briefly, in tabular form, for easy reference.

**Table 2:** Summary of additional criteria discussed in this review

	Topic	Take-home message
	Diameter	Good start—served us well
1	Pain	Pain mandates surgery
2	Length/tortuosity	A bit better than diameter
3	Genes	Powerful criterion
4	Family history	Powerful criterion
5	Bicuspid aortic valve	No longer a separate risk factor
6	Diabetes	Protective
7	Biomarkers (“RNA Signature” test)	Nearing validation
8	Aortic stress (exercise, blood pressure [BP])	Enter into decision-making “gestalt”
9	Root location of dilatation	Root more malignant than ascending
10	Inflammation on positron emission tomography (PET) imaging	PET “light-up” implies activity and rupture risk
11	*KIF6* gene	*KIF6* 719Arg variant increases risk nearly twofold
12	Sex	Raised risk in females, accommodated by body size indexing
13	Fluoroquinolone Rx	Dramatic increase in risk
14	Age	The aorta weakens as it ages, increasing the likelihood of aortic dissection at any given size
	Judgment	Key

Note: The criterion is named in the left column. The “snap” summary of the criterion is indicated in the right column. In the right column, text in **red** indicates a negative influence, text in **green** indicates a salutary influence, and text in **blue** indicates a neutral influence. Note (last row) our recommendation that despite scientific recommendations based on criteria 1–14, we recommend that clinical judgment be applied to put all component findings into a unified gestalt.

The clinician can apply added judgment along the axes discussed in this study to fine-tune the risk assessment for ascending aortic aneurysm patients—above and beyond that attainable solely by a diameter criterion. “Judgment” is the key word, as, despite decades of scientific investigation, the clinician's judgment is the key glue that assembles all the scientific clues outlined above into an organized gestalt for clinical application.
